# An improved pollen number counting method using a cell counter and mesh columns

**DOI:** 10.1186/s13007-020-00668-4

**Published:** 2020-09-14

**Authors:** Hiroyuki Kakui, Eriko Tsurisaki, Hidenori Sassa, Yoshinari Moriguchi

**Affiliations:** 1 Graduate School of Science and Technology, Niigata University, Niigata, Niigata, 950-2181 Japan; 2 Faculty of Agriculture, Niigata University, Niigata, Niigata, 950-2181 Japan; 3 Graduate School of Horticulture, Chiba University, Matsudo, Chiba, 271-8510 Japan

**Keywords:** Pollen number, Pollen size, CASY cell counter, Japanese cedar (*Cryptomeria japonica*)

## Abstract

**Background:**

The determination of pollen number is important in evolutionary, agricultural, and medical studies. Tree species of the Cupressaceae family cause serious pollinosis worldwide. Although Japanese cedar (*Cryptomeria japonica*) is the most important forestry species in Japan, it is also the biggest cause of pollinosis in the country. Japanese cedar trees have been selected for growth speed and superior morphological traits and then cloned. These clones may vary in their pollen production, but there has been little research on how many pollen grains are produced by a single male strobilus (flower). A recently reported method for counting pollen number with a cell counter was applicable to *Arabidopsis* species and wheat, but was not suitable for Japanese cedar because the strobilus does not open with heating (e.g. 60 °C, overnight).

**Results:**

Here, we report an improved pollen counting method for Japanese cedar using a precise and rapid cell counter in combination with home-made mesh columns. The male strobilus was gently crushed using a pestle. Large and small debris were then removed using 100- and 20-μm mesh columns, respectively. We successfully detected pollen sizes and numbers that differed between two clones using this method.

**Conclusions:**

This improved method is not only suitable for counting pollen from Japanese cedar, but could also be applied to other species of the Cupressaceae family with hard scale tissue covering the pollen. Moreover, this method could be applied to a broader range of plant species, such as wheat, because there is no need to wait for anthesis and debris can be removed efficiently.

## Background

The determination of pollen grain number is important in evolutionary, agricultural, and medical studies. From an evolutionary perspective, selfing plant species tend to produce lower pollen numbers than closely related outcrossing plant species [1–5]. The reduced pollen number in selfing plants is thought to decrease the cost of pollen production. From an agricultural perspective, domesticated species such as rice tend to have low pollen numbers [6]; however, the production of large numbers of pollen grains is one of the desired traits in hybrid wheat breeding [7]. A high pollen number is also a desired trait for crops that require artificial pollination because artificial pollination require a lot of pollen [8, 9]. From a medical perspective, pollen is relevant because it can lead to an allergic reaction called pollinosis [10, 11].

The recent development of next-generation sequencing techniques has enabled the genomic sequences of almost 600 plant species to be determined [12]. These sequenced genomes include those of plant species that have huge genome sizes, such as sugar pine (27.6 GB) or wheat (16 GB) [13, 14]. Combined analyses of sequence and phenotype data is a powerful tool for the identification of new genes. We recently identified a gene controlling pollen number using a genome-wide association study in *Arabidopsis thaliana* [5]. To estimate the pollen number from *A. thaliana* accessions, we developed a high-throughput method to count pollen grains efficiently [15]. Pollen numbers showed large variation within individuals and between species; therefore, the phenotyping step should be optimized based on the plant species and pollen number variation determined in a preliminary experiment.

The Cupressaceae family is conifer species with a world-wide distribution and it contains species causing serious pollinosis such as cypress (*Cupressus* species, *Chamaecyparis obtusa*), Japanese cedar (*Cryptomeria japonica*), and mountain cedar (*Juniperus ashei*) [16]. Japanese cedar is an evergreen tree and is the most important forestry species in Japan because it has excellent properties for use in Japanese architecture [17]. However, the pollen of Japanese cedar is the most serious allergen in Japan. Although the pollen grain number of Japanese cedar has been reported previously [18, 19], sample numbers were limited. Traditionally, the number of pollen grains has been counted using a hemocytometer under a microscope [20–22], but this method is time-consuming and laborious. Recently, efficient pollen counting methods using cell counter were developed [CASY cell counter (OMNI Life Science, Germany [15]) or Ampha Z32 (Amphasys, Switzerland [23, 24])]. Anthers from these plants were forcibly opened by heating (60 °C, overnight) from *A. thaliana* and wheat [15]. To count pollen grains of Japanese cedar using a cell counter, we attempted to apply the same protocol; however, the male strobilus (flower) of Japanese cedar consists of a hard scale structure and it did not open with heating. Here, we report an improved pollen counting protocol using a cell counter and home-made mesh columns. We confirmed that Japanese cedar pollen can be counted efficiently using this method.

## Methods

### Plant materials

Trees of Japanese cedar (*Cryptomeria japonica*) were grown in the Niigata prefectural forest research institute. Three clones, ‘Iwafune-9’, ‘Iwafune-15’, and ‘Nishikanbara-1’ were used. Male strobili were collected in February 2020. Pollen grains are already matured during this period [25].

### Preparation of home-made mesh columns

**Fig. 1:**
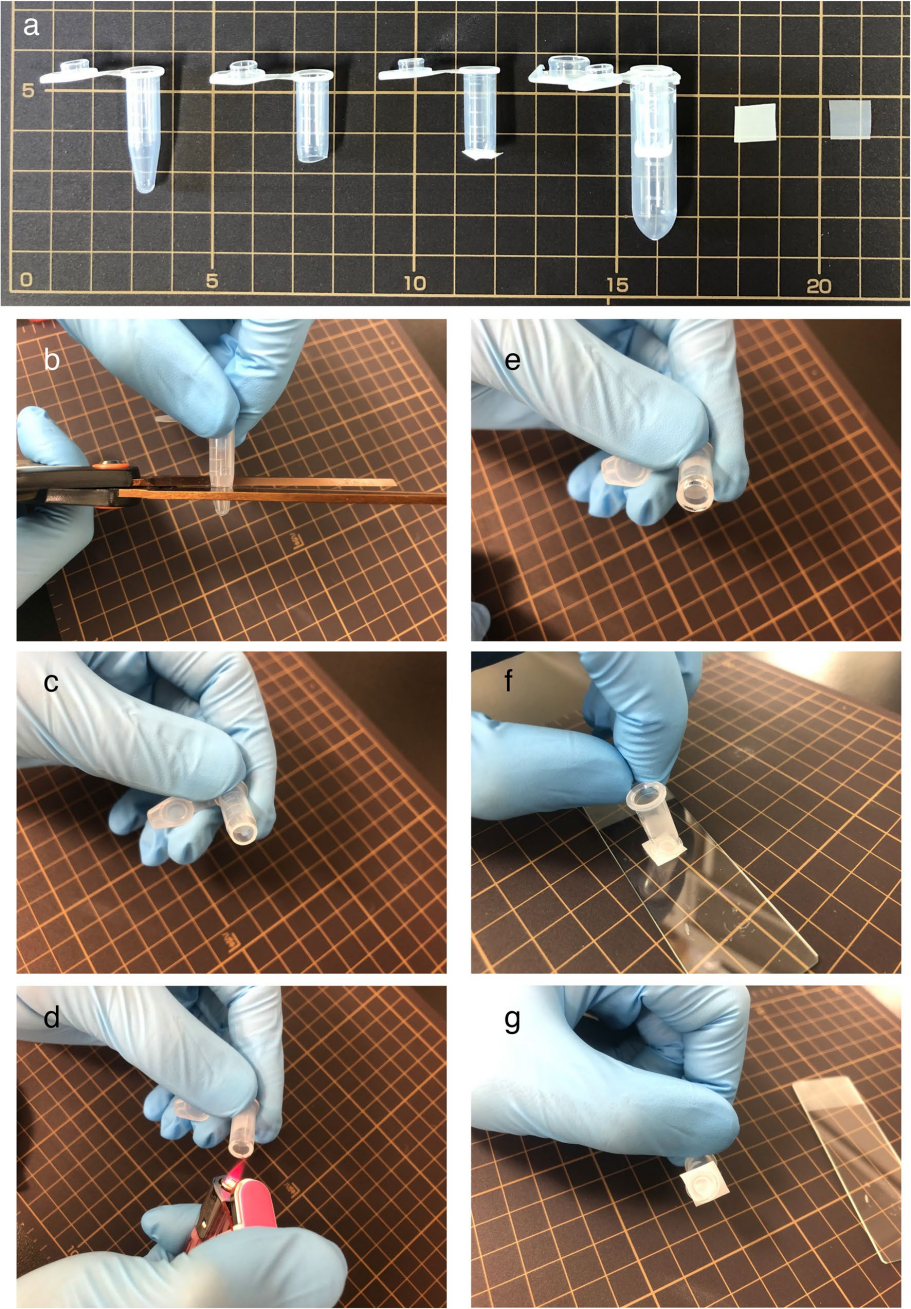
Home-made mesh columns. **a** Left to right: 0.6-mL tube, 0.6-mL tube with cut tip, mesh attached to 0.6-mL tube (column), and polyester meshes (20-μm and 100-μm opening sizes). **b–g** Column preparation. The bottom of a 0.6-mL sample tube was removed around the 100-μL line using scissors (**b** and **c**). The cut surface was heated using a cigarette lighter (**d** and **e**). The heated surface was bound with 1 × 1 cm pieces of polyester mesh (**f** and **g**). The background squares were 1 × 1 cm

To remove large and small debris, two types of polyester mesh [20-μm opening size (MEDIFAB 07-20/13, Sefar AG, Switzerland) and 100-μm opening size (PETEX PET105, Sefar AG)] were used. The concept of using mesh columns was derived from a protein experimental protocol [26]. The bottom of a 0.6-mL sample tube was removed around the 100-μL line using scissors and bound with the polyester mesh by heating (for details see Fig. 1). The pieces of mesh were 1 × 1 cm (Fig. 1).

### Preparation of pollen suspension

**Fig. 2:**
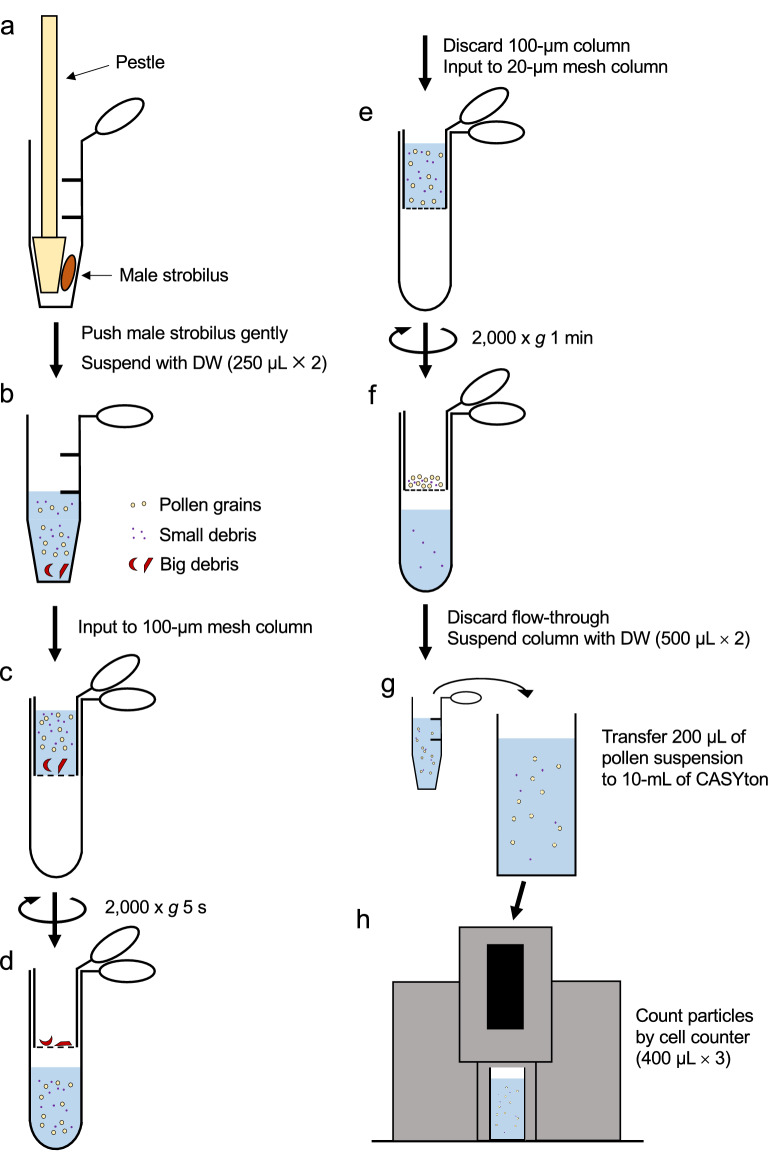
Flowchart of the pollen grain counting technique. Outline of the protocol and the corresponding procedure steps. **a** Male strobilus is crushed gently in a 1.5-mL tube and suspended by DW (250 μL × 2). **b–d** Pollen suspension is transferred to 100-μm mesh column and centrifuged to remove bigger debris. **e**, **f** The flowthrough is transferred to a 20-μm mesh column and centrifuged to remove small debris. **g** Pollen-containing particles are mixed with water again (500 μL × 2). Part of pollen suspension (200 μL of 1 ml) is mixed with 10 mL of CASYton. **h** Particle numbers are counted by a cell counter

**Fig. 3:**
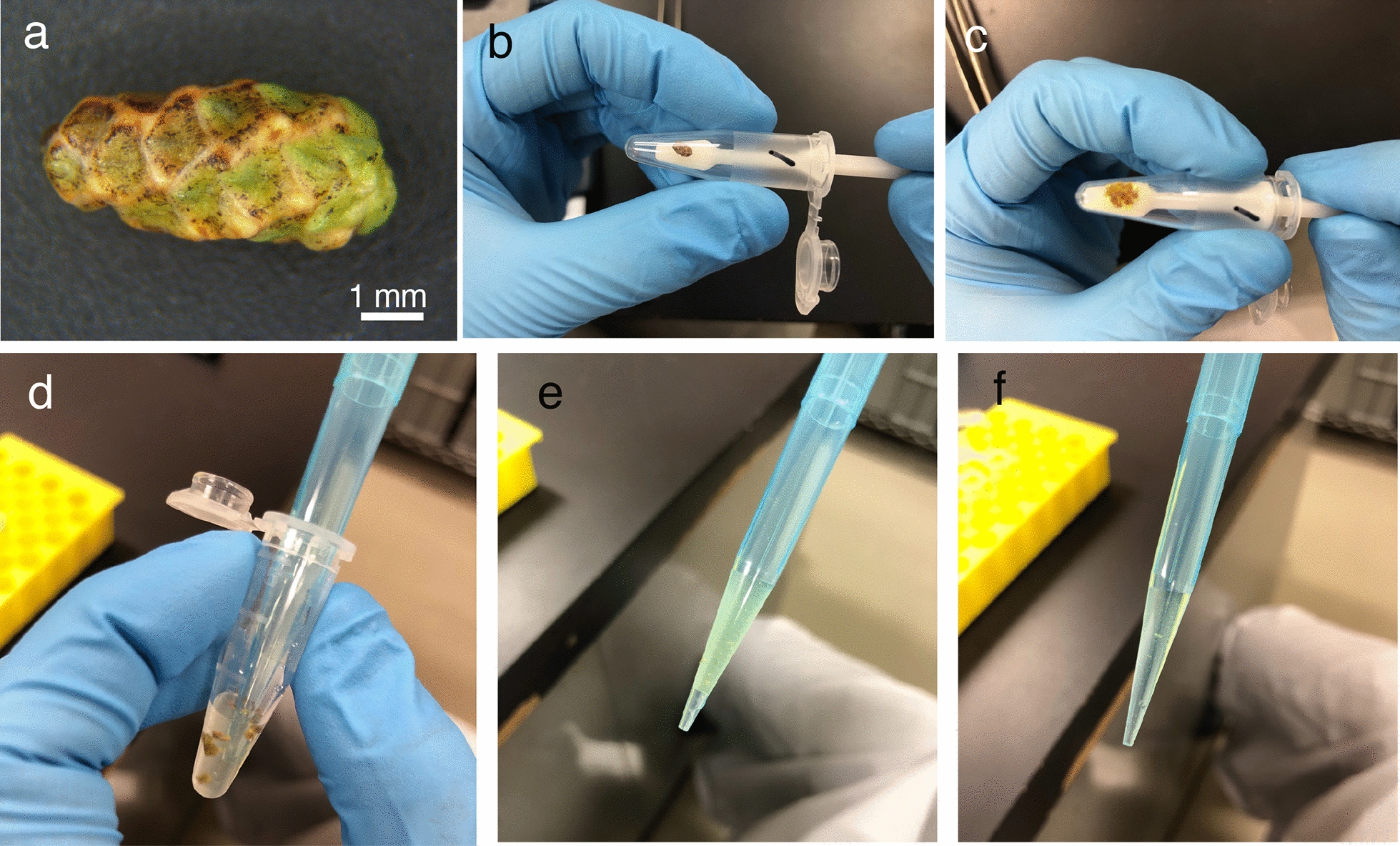
Crushing and collecting pollen-containing liquid from male strobilus. **a** Male strobilus of Japanese cedar. Pollen grains were covered by a hard scale structure. Bar indicates 1 mm. **b**, **c** Male strobilus crushed by a pestle. **d–f** Pollen-containing suspension. Pollen-containing particles were collected from two suspensions (**e** and **f**)

A flowchart of the pollen suspension preparation method is provided in Fig. 2. The male strobilus was gently crushed using a pestle in a 1.5-mL tube and 250 μL of distilled water (DW) was added to the 1.5-mL tube (Figs. 3a–d). The pollen-containing suspension was moved to a new 1.5-mL tube. To collect almost all of the pollen, an additional 250 μL of DW was added to the first tube and remaining pollen was suspended. This suspension was then added to the first pollen suspension for a total volume of 500 μL. The suspension was transferred to a 100-μm mesh column and centrifuged at 2000*g* for 5 s. The column retaining the large male strobilus tissues was discarded and the flowthrough was transferred to a 20-μm mesh column. This suspension was centrifuged at 2000*g* for 1 min. Particles > 20 μm that were trapped by the mesh were suspended with 500 μL of DW and transferred to a new 1.5-mL tube. The 20-μm mesh column was washed with another 500 μL of DW to collect almost all of the remaining particles and transferred to the 1.5-mL tube for a total volume of 1 mL.

### Pollen counting using a CASY cell counter

The cell counter (CASY cell counter) parameters were set as described in Table 1. We chose the size range from 27.75 to 45 μm as pollen particle because this range covered pollen peak from all samples without contamination from small/large particles. A 200-μL pollen suspension was mixed with 10 mL of CASYton (OMNI Life Science). Particle numbers were counted using a cell counter by sampling 400 μL three times. Viable cells were calculated as the total pollen number per strobilus using the following equation:

$$\begin{aligned} {\text{Total}}\;{\text{pollen}}\;{\text{number}}\;{\text{per}}\;{\text{flower}} \\ { = } & \;{\text{Viable}}\;{\text{cell}}\;{\text{number}}\;{\text{counted}}\;{\text{by}}\;{\text{cell}}\;{\text{counter}} \\ \times & \;\frac{{{\text{Total}}\;{\text{suspension}}\;{\text{volume}}\;{\text{applied}}\;{\text{to}}\;{\text{cell}}\;{\text{counter}}\; ( 1 0. 2\;{\text{ml)}}}}{{{\text{Suspension}}\;{\text{volume}}\;{\text{counted}}\;{\text{by}}\;{\text{cell}}\;{\text{counter}}\; ( 1. 2\;{\text{ml)}}}} \\ \times & \;\frac{{{\text{Total}}\;{\text{pollen}}\;{\text{suspension}}\; ( 1\;{\text{ml)}}}}{{{\text{Pollen}}\;{\text{suspension}}\;{\text{mixed}}\;{\text{with}}\;{\text{CASYton}}\; ( 0. 2\;{\text{ml)}}}} \\ \end{aligned}$$

Taken together, cell counter counted 1/42.5 of pollen grains of one strobilus.

**Table 1:** Parameter for counting pollen number of Japanese cedar

Capillary size	150 μm
Size scale	50 μm
Range: debris	< 20 μm
Range: dead cells	20–27.74 μm
Range: viable cells	27.75–45 μm
Sample volume	3 × 400 μL

### Data analysis

The distribution of pollen number and size data from a single strobilus were displayed using the CASY application (OMNI Life Science). A scatter plot was constructed using the R package ggplot2 [27].

## Results

### Crushing the male strobilus using a pestle to collect pollen grains

To count the number of pollen grains per strobilus precisely, it is important to collect all of the pollen grains from the strobilus. The model plant *A. thaliana* has thin anthers that open easily at high temperatures (60 °C, overnight [15]). We attempted to open the male strobili of Japanese cedar by heating or drying, but this approach was not successful. The male strobilus of Japanese cedar has a hard scale structure, unlike the anthers from *Arabidopsis* species or wheat [15] (Fig. 3a). We then attempted to crush the male strobilus with a pestle to break the scale structure. Pollen grains are physically stronger than other plant tissues because they have an external wall layer called the exine, which is a physically and chemically resistant structure [28]. A male strobilus was gently crushed using a pestle (Fig. 3b, c), and then suspended in DW using a pipette (Fig. 3d). The scale structure was separated after pipetting and we successfully collected all of the pollen from the pollen-containing suspension (Fig. 3e, f).

### Removing large debris from the pollen-containing suspension using a 100-μm mesh column

The pollen suspension contained not only pollen grains but also large amounts of large and small debris (e.g., scale tissue, anther wall, etc.). Because the cell counter cannot count particles > 150 μm due to the capillary becoming blocked, we made a 100-μm mesh column to remove the large debris. Japanese cedar pollen typically has a round shape with a 35-μm diameter as determined from microscope observations so it was unlikely to be retained by the 100-μm mesh column. The pollen-containing suspension was loaded onto the 100-μm mesh column and centrifuged. Large scale structures were retained in the column without stopping the passage of the pollen grains. Pollen grains moved to the flowthrough area (Fig. 2d). The capillary of the cell counter remained unblocked throughout this study, suggesting that the 100-μm mesh column successfully removed large debris.

### Removing small debris from the pollen-containing suspension using a 20-μm mesh column

**Fig. 4:**
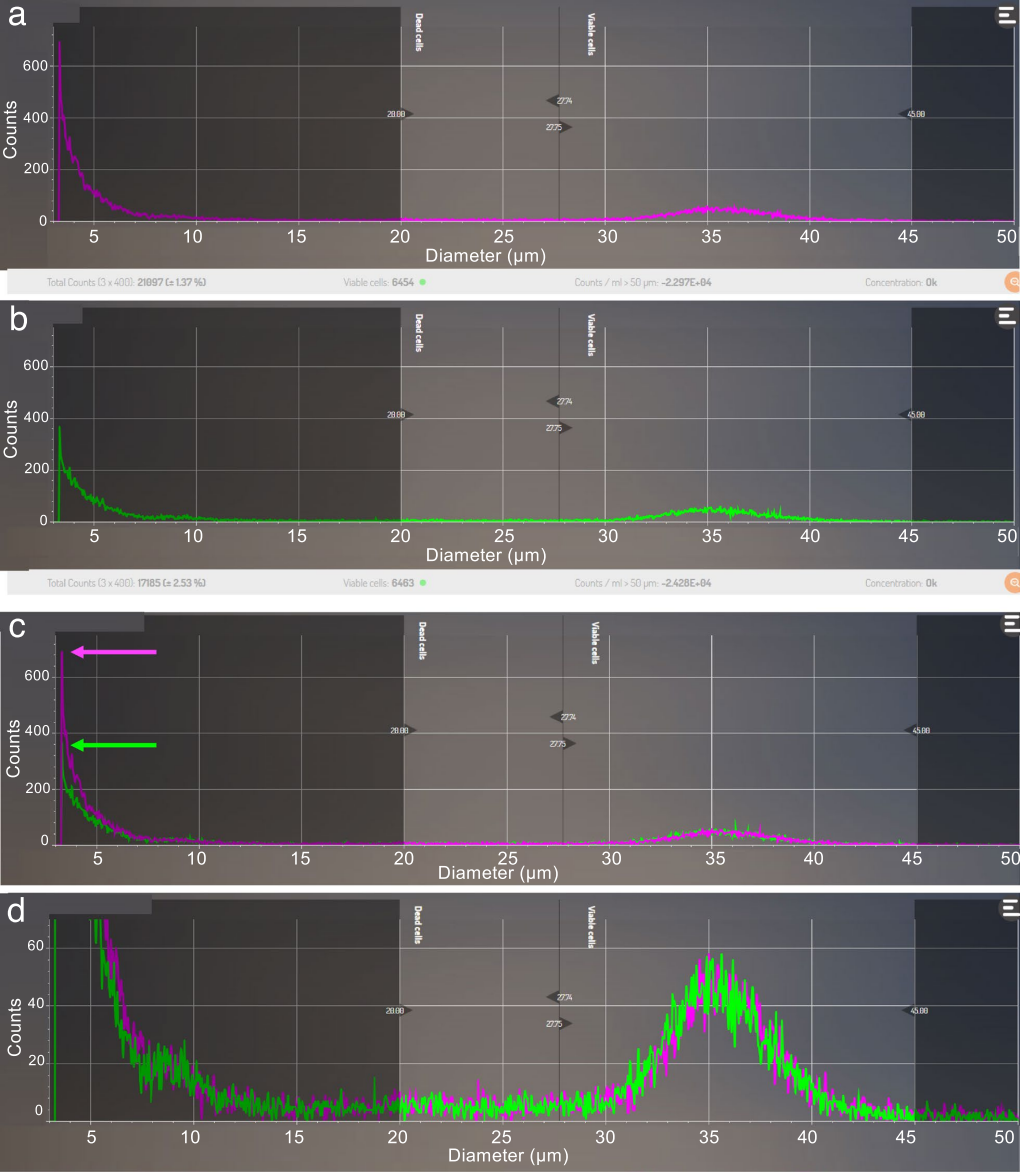
Removal of small particles using a 20-μm mesh column. Particle distributions from identical samples of ‘Iwafune-15’ clone were determined without (**a** and **c**; magenta) and with (**b** and **c**; green) passage through the 20-μm mesh column. Merged sample data are shown in **c** and **d**. The X axis indicates particle diameter (μm) and the Y axis indicates particle count. The limit of the Y axis is 750 counts in **a–c** and 70 counts in **d**. Peaks of the small debris section (0–20 μm) are indicated by magenta (without 20-μm mesh column) and green (with 20-μm mesh column) arrows in **c**

**Fig. 5:**
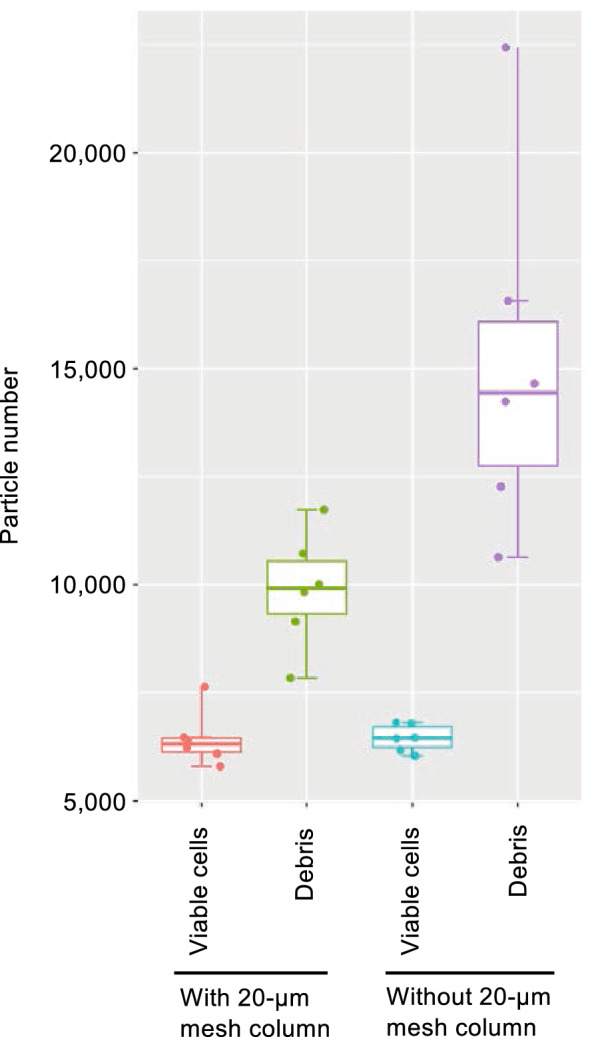
Pollen and debris number with or without passage through the 20-μm mesh column. Particle numbers of pollen grain and debris were shown by boxplots. Boxplots were made by Table 2 data. N = 6, 6, 6, 6. Boxplots show center line: median; box limits: upper and lower quartiles; horizontal line of whiskers: not greater than 1.5 times the interquartile range

**Fig. 6:**
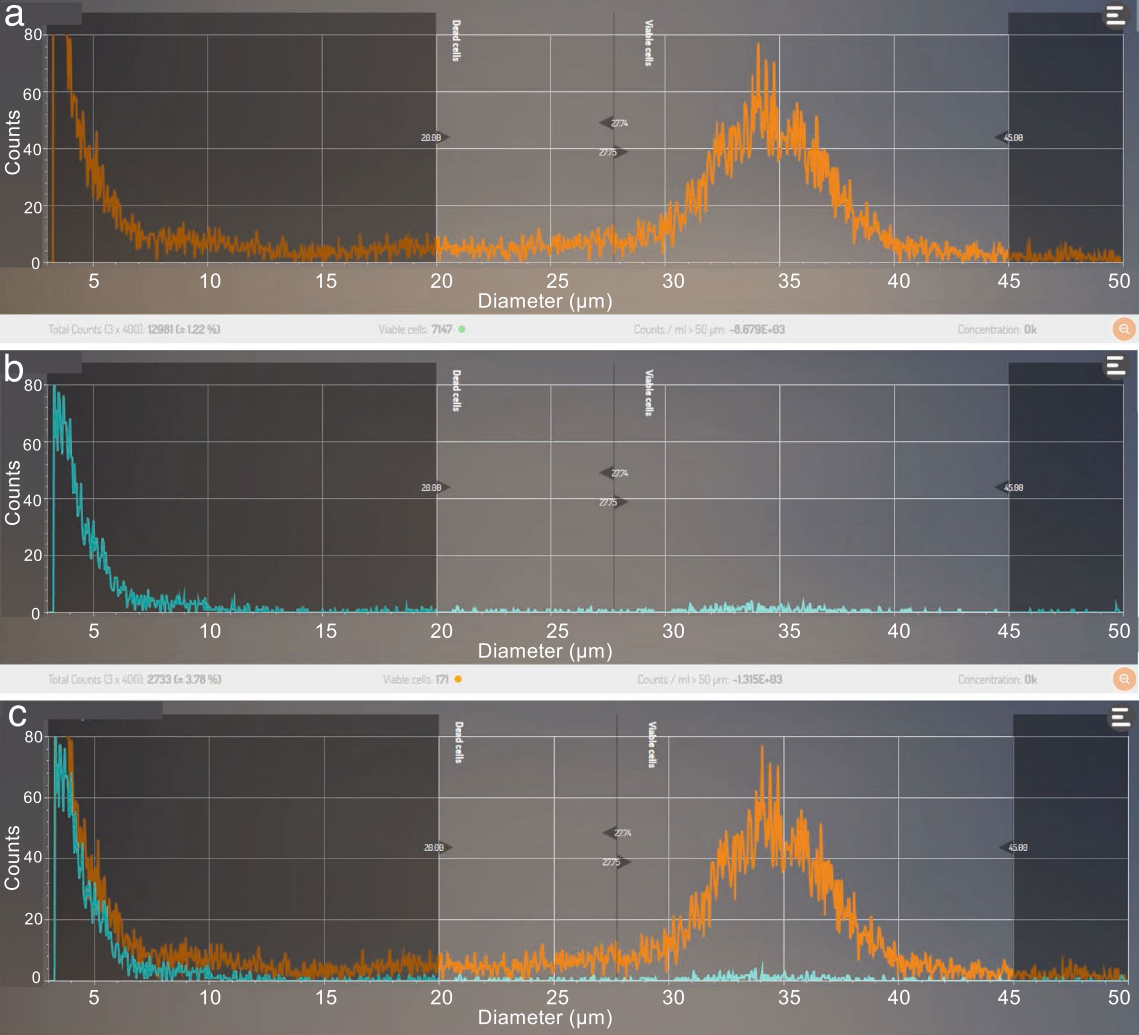
Particle distribution from the flowthrough liquid after using the 20-μm mesh column. Particle distributions in the pollen suspension (**a**) and flowthrough section (**b**) from ‘Iwafune-15’ clone. There was almost no detectable peak from the flowthrough section (**b** and **c**)

The cell counter can count a maximum of 20,000 particles in a single measurement. The flowthrough suspension passing through the 100-μm mesh column contained many small particles. Figure 4 shows the particle distribution with or without passage of the same sample through a 20-μm mesh column. More than 20,000 particles were detected in a pollen suspension without passage through a 20-μm mesh column (total of 21,097 particles; Fig. 4a). In contrast, many small debris particles were removed after using the 20-μm mesh column (total of 17,185 particles remained in suspension; Fig. 4b, c). In the 27.75- to 45-μm particle size range, almost the same number of pollen grains remained with or without the use of the 20-μm mesh column (6454 vs 6463 particles, respectively; Fig. 4d). Table 2 and Fig. 5 shows the efficiency of the 20-μm mesh column for removing small debris. Approximately, 32% of the debris was removed using the 20-μm mesh column. To check whether pollen grains leaked through the 20-μm mesh column, the pollen number was counted for the pollen retained by the 20-μm mesh (normal pollen suspension) and for the pollen in the flowthrough after using the 20-μm mesh column. The particle distribution pattern revealed a clear pollen peak from the pollen sample retained by the mesh, whereas there was no peak observed in the flowthrough (Fig. 6). Table 3 shows the numbers of particles between 27.75 and 45 μm in the 20-μm mesh column-trapped samples and flowthrough samples. Less than 3% of the particles were detected in flowthrough samples for all samples. These results suggest that the use of the 20-μm mesh column reduced the amount of small debris in the pollen suspension with no loss of pollen grains.

**Table 2:** Particle numbers with or without passage through the 20-μm mesh column

Sample No.	Clone name	Sample with 20 μm mesh column			Sample without 20 μm mesh column			With/without 20 μm mesh column		
		Total counts	Viable cells	Debris	Total counts	Viable cells	Debris	Reduced debris number	Reduced ratio (%)	Viable cell ratio (%)
1	Iwa-9	15237	6094	9143	19072	6806	12266	3123	25.5	89.5
2	Iwa-15	17526	5797	11729	20266	6040	14226	2497	17.6	96.0
3	Iwa-15	16235	6222	10013	28594	6165	22429	12416	55.4	100.9
4	Iwa-15	17451	7633	9818	23346	6786	16560	6742	40.7	112.5
5	Iwa-15	17185	6463	10722	21097	6454	14643	3921	26.8	100.1
6	Iwa-15	14232	6394	7838	17065	6433	10632	2794	26.3	99.4
							Average	5249	32.0	99.7

**Table 3:** Pollen numbers from 20-μm mesh column-trapped sample and flow through sample

Sample no.	Viable cells counts		Viable cell ratio (%)
	20 μm column	Flow through	(Flow through/Total counts)
1	4597	133	2.81
2	4563	121	2.58
3	4381	118	2.62
4	5378	130	2.36
5	3826	44	1.14
6	6233	179	2.79
7	6578	171	2.53
8	5446	154	2.75
9	7421	193	2.53
10	7147	171	2.34
		Average	2.45

### Numbers and sizes of pollen grains from two Japanese cedar clones

**Fig. 7:**
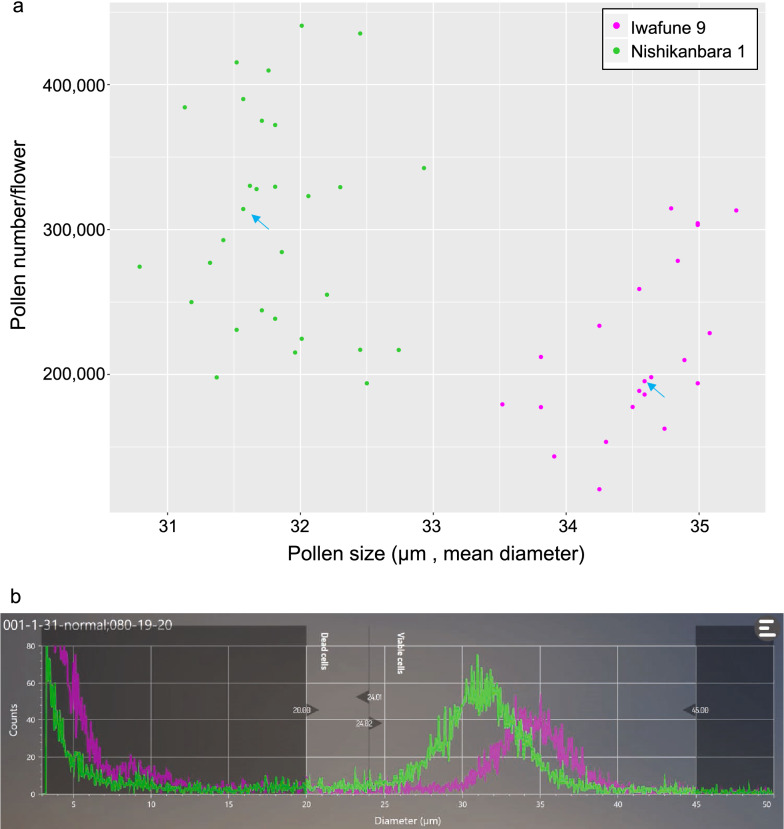
Pollen numbers and size distributions from two Japanese cedar clones. **a** Scatter plots of ‘Iwafune-9’ (magenta) and ‘Nishikanbara-1’ (green) clones. Each dot indicates pollen number and size data from a single strobilus. Blue arrows indicate the representative samples of each clone displayed in **b**. **b** Particle distributions from representative samples

Pollen number and size for ‘Iwafune-9’ and ‘Nishikanbara-1’ were measured by the cell counter. Twenty-two strobili from ‘Iwafune-9’ and 30 strobili from ‘Nishikanbara-1’ were analyzed. Figure 7a shows the pollen number and size distribution of 52 samples. The two clones had clearly different pollen sizes. ‘Iwafune-9’ had a low pollen grain number (mean pollen number = 196,754) but a larger pollen size (mean pollen diameter = 34.59 μm) compared to ‘Nishikanbara-1’ (mean pollen number = 304,429, mean pollen diameter = 31.79 μm). Even among clones, there was more than a two-fold difference in pollen number. Such a large variation in pollen number from the same plant has also been reported in *Arabidopsis* species and in wheat [5, 15]. Figure 7b shows the particle size distribution for representative samples from ‘Iwafune-9’ (magenta) and ‘Nishikanbara-1’ (green). Both samples showed a clear single peak in the viable cell range, which is the size range we expected based on microscopy observations. There was typically a 10-μm variation in pollen size within the same strobilus (e.g., 30 to 40 μm from ‘Iwafune-9’ and 26 to 37 μm from ‘Nishikanbara-1’; Fig. 7b).

### Detection of pollen cells released from exines using a cell counter

**Fig. 8:**
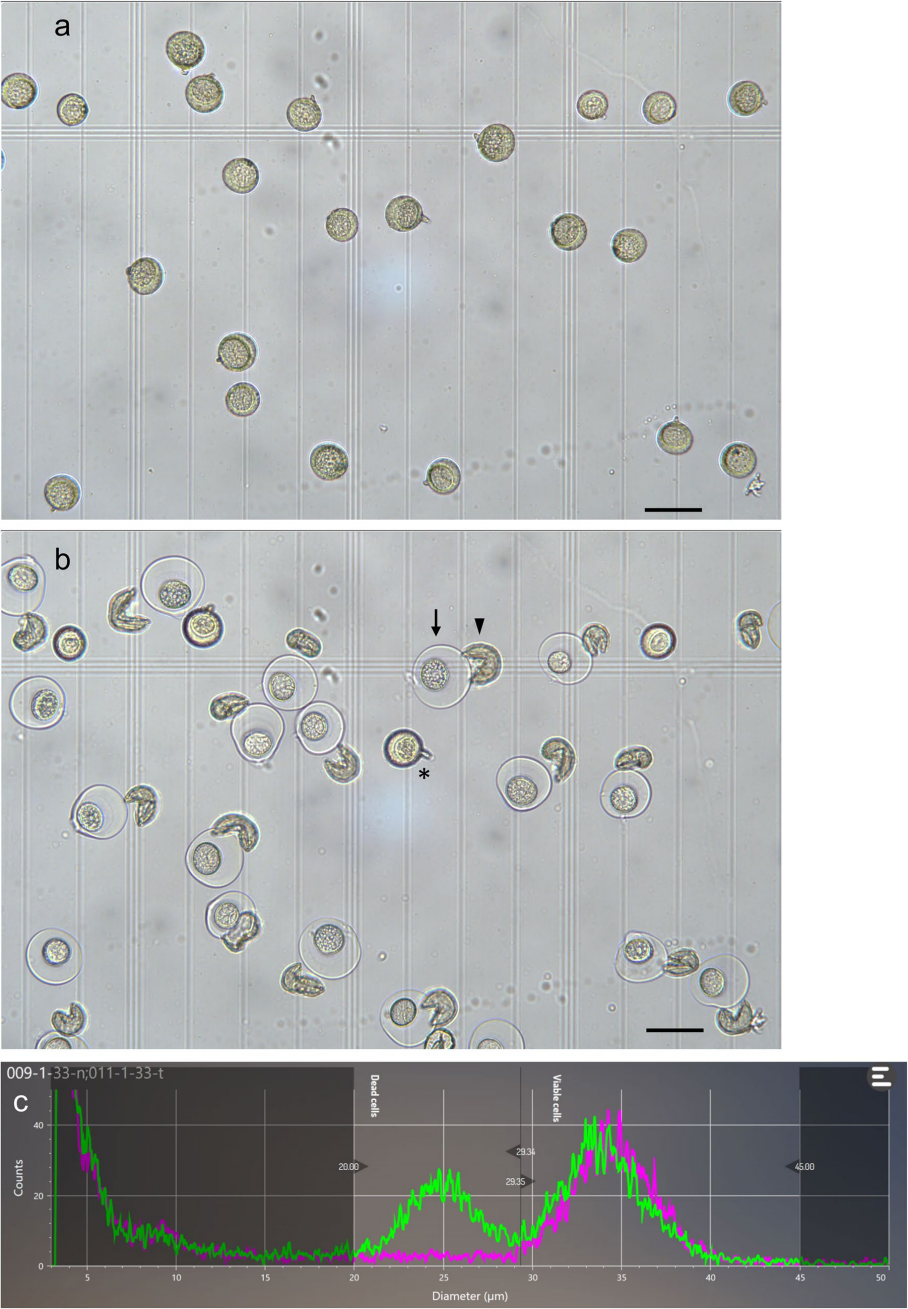
Artificial pollen release and detection by the cell counter. Microscope observations of pollen grains from ‘Iwafune-9’ clone in CASYton at 0 min (**a**) and 30 min (**b**). Eighteen of the 22 pollen grains had released the pollen cell from the exine structure after 30 min. Bars = 50 μm. Arrow: released pollen cell, arrowhead: exine structure, asterisk: unreleased pollen. **c** Particle distributions at 0 min (magenta) and 30 min (green) for the same strobilus sample. Additional peaks derived from removed exine were observed in the sample after 30 min

Pollen of Japanese cedar has some unique features compared with angiosperm plant species. For example, mature Japanese cedar pollen cells include generative cells and tube cells [29]. When mature pollen attaches to the nucellus (pollination), the intine structure, including the pollen cell, is released from the exine structure and the germinated pollen tube grows through the nucellus [29]. This process is important for pollinosis patients because Cry j1 and Cry j2, which are the major allergenic proteins of Japanese cedar, are localized in the intine and intine is also released from the exine in the human eye [30–32]. In this study, most of the pollen grains were not released from exines after 24 h in DW (data not shown). On the other hand, we found that many pollen grains were released from the exine structure in CASYton after 30 min (Fig. 8a, b). The cell counter displayed an additional small peak after the pollen suspension was mixed with CASYton (Fig. 8c). The original pollen peak remained almost the same between 0 and 30 min. Although the two peaks derived from the exine and pollen cells were indicated by different particle diameters, we recommend determining the pollen number immediately after mixing with CASYton for species which have similar traits because a small overlap of the two peaks was detected (Fig. 8c, around 30 μm diameter).

## Discussion

### Effectiveness of the improved pollen counting protocol with home-made mesh columns

We developed an improved protocol that allows pollen grains to be collected from a single strobilus without waiting for anthesis. We also demonstrated that two types of mesh could remove large and small debris efficiently. This system is a cost-effective method because the columns are home-made (one column costs less than 50 cents). In this method, the cell counter can count 1/42.5 of pollen grains of single strobilus within 5 min. This is much efficient and faster than the traditional microscope method because the standard microscope method only counts 1/10,000 of pollen grains of single flower/strobilus in 17 min. [15]. Using this method, we can make lower pollen number clones by crossing low pollen number parents with superior woody traits. These offspring will have lower pollen numbers but have fine woody traits. For clonal propagation such as grafting, efficient pollen number counting will also contribute to choosing lower pollen number clones.

The cell counter revealed the pollen number and size variation between clones/samples and within samples in Japanese cedar. There was a 1.5-fold difference in pollen number between the two clones. The clones were clearly distinguishable by pollen size. Broader pollen measurements using more clones will reveal trends in pollen number and size in Japanese cedar in the future.

This column method is applicable not only for Japanese cedar but is also possibly applicable to a broader range of species. For example, the strobili of Cupressaceae species, such as *Cupressus*, *Juniperius*, and *Chamaecyparis* species, typically have scale tissue similar to the Japanese cedar [33]. Counting the pollen grain number of these species is important because they are also pollinosis-causing species worldwide [16, 34, 35]. In plant breeding, an increase in pollen number is a desired trait [7]. Although we previously established a pollen counting method for wheat, elongation of anther filaments takes time and the method requires cutting both tips of the anther with a syringe to enhance pollen release [15]. The mesh column method described in this paper is less labor intensive and we are adopting this method to count wheat pollen now. In summary, this column method could possibly be applied to a broader range of species. The recent development of next-generation sequencing techniques allows unique genes to be identified in non-model species [36, 37]. Whole-genome sequencing is proceeding in Japanese cedar. The combination of our improved pollen counting method with a genome-wide analysis will provide new insights into pollen number, such as the identification of pollen number-controlling genes from Japanese cedar and wheat.

## Conclusions

Herein, we report the efficient, high-throughput, and cost-effective pollen grain counting method applicable to flowers with hard scale tissue. This method is able to count 20,000 particles within 5 min. It is more than 100 times than traditional hemocytometer method. Two types of home-made column work to remove large or small particles effectively at low cost. This method is not only applicable to Japanese cedar but also to a broad range of plant species.

## Data Availability

The datasets used and/or analysed during the current study are available from the corresponding author on reasonable request.

## Abbreviation

DW: Distilled water
